# Proposal of kHz-level polarization switching of undulator radiation with magnetic field modulation at Hefei Advanced Light Facility

**DOI:** 10.1107/S1600577526006533

**Published:** 2026-07-27

**Authors:** Nanrui Yang, Junce Yang, Zhouyu Zhao, Heting Li

**Affiliations:** a National Synchrotron Radiation Laboratory University of Science and Technology of China Hefei Anhui20029 People’s Republic of China; SESAME, Jordan

**Keywords:** undulator radiation, fast polarization switching, field modulation

## Abstract

A magnetic field modulation-based scheme for fast polarization switching of undulator radiation has been proposed for Hefei Advanced Light Facility (HALF), a fourth generation soft X-ray light source based on a diffraction-limited storage ring. In this scheme, two undulators with opposite helicities are employed, each equipped with electromagnetic coils placed on the upper and lower surfaces of the vacuum chamber. Through fast switching the power of the coils, the radiation frequencies of two undulators can be rapidly shifted into and out of the band pass of a monochromator; polarization switching at the kHz level in the photon energy range 330–1000 eV for user beamlines can be expected with a stainless steel vacuum chamber. Crucially, a vacuum chamber designed for HALF is selected to be used for the simulations, representing the first demonstration of achieving such switching frequency under realistic vacuum chamber conditions. The limitations imposed by chamber-induced eddy currents have been studied. Benefiting from the low current and compact coil size, polarization switching at approximately 100 Hz can still be achieved even with an aluminium or copper vacuum chamber. Numerical simulations confirm the feasibility of the scheme, demonstrating high polarization degree and maintained high photon flux. Furthermore, these coils are designed to correct the first and second integrals of magnetic field themselves. This method can also be easily adopted for other light sources requiring fast polarization control.

## Abstract 2

This paper presents a proposal of kHz-level fast polarization switching of undulator radiation with magnetic field modulation using electromagnetic coils at Hefei Advanced Light Facility.

## 1. Introduction

In recent years, various types of undulators, such as the variable-polarization undulators APPLE-II (Sasaki *et al.*, 1993 ▸; Sasaki, 1994 ▸) and APPLE-X (Schmidt & Calvi, 2018 ▸; Liang *et al.*, 2021 ▸), harmonic-suppressing quasi-periodic undulators (Sasaki, 2010 ▸), and figure-8 undulators (Tanaka & Kitamura, 1995 ▸; Tanaka *et al.*, 1999 ▸), have been developed to enable precise tailoring of undulator radiation characteristics, including polarization degree, flux distribution and photon energy. Building upon these advancements, fast polarization switching is becoming increasingly important for experiments such as XMCD/XMLD (X-ray magnetic circular/linear dichroism) to investigate the orbital and spin moments of materials. To achieve fast polarization switching, various methods have been developed, primarily focusing on two strategies: direct control of the magnetic source and selection of the photon beam. The most direct approach involves innovating the magnet structure itself, by mechanically adjustable undulators, such as the APPLE-type undulators, which can control the polarization states by shifting magnetic arrays. Hybrid permanent magnet-electromagnet designs, implemented at SOLEIL (Marteau *et al.*, 2009 ▸; Marteau *et al.*, 2011 ▸) and ESRF (Chubar *et al.*, 2004 ▸), enable fast polarization switching of circular polarization by reversing the coil current. When the magnetic source itself cannot be altered fast enough, ‘beamline switching’ offers another path to achieve fast polarization switching. This is typically realized using twin-undulator systems, where two undulators generate radiation with different polarization states. Techniques such as electromagnetic switching (Tanaka *et al.*, 2002 ▸; Sánchez-Hanke *et al.*, 2009 ▸), photon beamline switching (Sawhney *et al.*, 1997 ▸; Weiss *et al.*, 2001 ▸; Bahrdt *et al.*, 2001 ▸; Schmidt *et al.*, 2001 ▸), electron beam orbit switching (Hara *et al.*, 2003 ▸; Cao *et al.*, 2016 ▸; Tsuchiya *et al.*, 2013 ▸) or natural closed-orbit switching (Holldack *et al.*, 2020 ▸) are then employed to select the desired polarized photon beam, effectively enabling ‘alternating’ delivery of one polarization at a time.

Furthermore, an innovative approach based on ‘spectral modulation’ provides a third strategy. Instead of physically switching the polarization, this method introduces phase shifters between undulators (Kim, 1984 ▸; Tanaka & Kitamura, 2002 ▸; Yamamoto *et al.*, 2014 ▸; Kinjo & Tanaka, 2016 ▸; Yang *et al.*, 2023 ▸) to split the spectra or applies a modulated magnetic field to slightly detune the undulator harmonics (Rowen & Lüning, 2001 ▸; Holldack *et al.*, 2020 ▸; Kesgin *et al.*, 2019 ▸; Yang *et al.*, 2024 ▸). Cross undulators are another scheme that achieves fast polarization switching with the help of phase shifters (Kim, 1984 ▸; Bahrdt *et al.*, 1992 ▸; Tanaka & Kitamura, 2004 ▸; Matsuda *et al.*, 2014 ▸; Kaneyasu *et al.*, 2020 ▸; Miyawaki *et al.*, 2021 ▸). The first polarization switching demonstration using crossed-planar undulators in a seeded free-electron laser was carried out at Shanghai (Deng *et al.*, 2014 ▸).

In our previous work, an alternative polarization switching scheme based on magnetic field modulation was proposed and preliminarily investigated (Yang *et al.*, 2024 ▸). This scheme offers significant advantages, such as collinear emission of a radiation beam in two different polarization states, while the electron beam trajectory remains highly stable. Furthermore, the scheme ensures the generation of high-quality radiation, achieving both high polarization and high photon flux simultaneously, remaining strong engineering feasibility.

Hefei Advance Light Facility (HALF) is a 2.2 GeV diffraction-limited storage ring, developed by National Synchrotron Radiation Laboratory in China (Wang *et al.*, 2018 ▸; Bai *et al.*, 2021 ▸; Zhao *et al.*, 2023 ▸). It has 20 long straight sections and 20 middle straight sections. A specialized undulator system has been utilized to realize fast polarization switching. Two EPU41 will be installed sequentially and connected by a phase shifter in one straight section.

In this paper, we further investigate the feasibility of upgrading the existing polarization-switching scheme for HALF with magnetic field modulation. Based on the parameters of the planned fast polarization-switching undulators and the vacuum chamber designed for the HALF straight section, we propose a novel coil design and conduct comprehensive simulations of its performance. The rest of this paper is organized as follows. First, a theoretical analysis of the proposed scheme is presented based on an electron radiation model. Then, the methods of the coil design are studied in detail. Additionally, the radiation performance with modulating coils, constraints imposed by coil inductance and eddy current effect of the vacuum chamber are evaluated and discussed. Finally we provide a summary.

## 2. Basic design

### 2.1. Polarization switching by field modulation

Let us recall the spectrum of the undulator radiation. To illustrate the basic principle of the proposed scheme, in the following we introduce an electron radiation model to accurately calculate the photon energy shift generated by the magnetic field of the coils. When an electron beam passes through an undulator with modulating field, the vertical magnet field *B*_*y*_ that it experiences can be expressed as the superposition of the undulator field and the weak coil field. The horizontal magnet field *B*_*x*_ is the same as the field of a conventional undulator without field modulation. 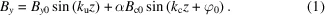
Here, *k*_u_ is 2π/λ_u_ with λ_u_ being the period of the undulator, *k*_c_ is 2π/λ_c_ and λ_c_ is the period of the modulation field, or the coils. The factor α = *B*_c0_/*B*_*y*0_ is the intensity factor of the coil field, representing the ratio of the peak field of the coil to *B*_*y*0_. The period of the coil field is *m* times that of the undulator. *B*_*y*0_ is the peak field of the undulator in the vertical direction and *B*_*c*0_ is the peak field of the coil. φ_0_ is the phase deviation between the coil field and the undulator field, corresponding to the initial position of the weak field relative to the main field. Calculating the horizontal velocity of electrons, and using *K*_*y*_ = 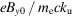, the undulator strength parameter in the vertical direction, the average velocity of electrons along the undulator direction in a complete period λ_c_ can be deduced. Using the undulator emitting model one can obtain the fundamental energy of on-axis radiation,
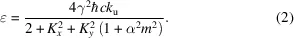
Here, *m* = *k*_u_/*k*_c_ = λ_c_/λ_u_, which means that the period of the coil field is *m* times that of the undulator. *K*_*x*_ is the undulator strength parameter in the horizontal direction and ℏ is the reduced Planck constant. Focusing on the fundamental radiation, using *K* = 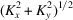, only when the photon energy shift induced by the coil field is large enough can the radiation flux after passing through the monochromator be reduced to an extremely low level. Considering the undulator radiation bandwidth, the required shift of the photon energy can be described as Δɛ/ɛ ≥ 1/*N*_u_, with *N*_u_ being the number of periods of the undulator, then one can obtain the requirement on the product of the weak field and the coil period, 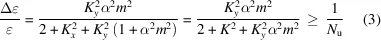

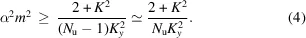
This relation reveals a significant design insight: employing a coil with a longer period (larger *m*) can drastically reduce the required magnetic field strength α to achieve the same energy shift. The basic principle of the proposed scheme has been illustrated in detail in our previous work (Yang *et al.*, 2024 ▸).

**Figure 1:**
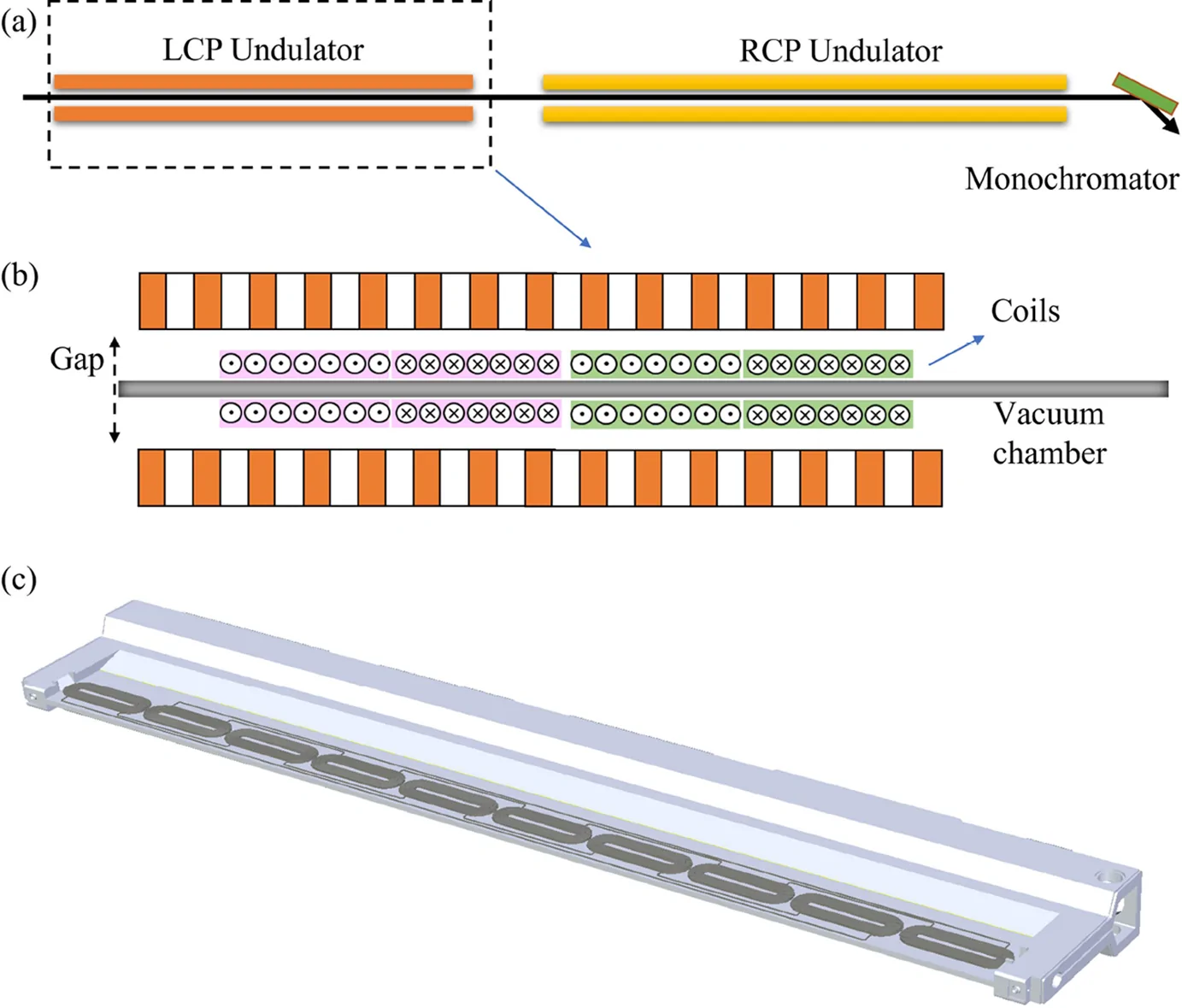
(*a*) Schematic of the setup of the device. (*b*) Field modulation undulator. (*c*) Setup of the coils and vacuum chamber used in the simulations.

Now, let us explain how to switch the helicity of circular polarization through the scheme described above. As illustrated in Fig. 1 ▸(*a*), two identical undulators are positioned on the same beamline, operating in different polarization states to emit radiation with opposite polarization states, for example, right-hand and left-hand circularly polarized (RCP and LCP, respectively). For fast polarization-switching applications, APPLE-II undulators or EPUs are commonly employed. A monochromator is positioned downstream, with each undulator incorporating dedicated electromagnetic coils. The modulation coils are installed on the upper and lower surfaces of vacuum chamber [Figs. 1 ▸(*b*), 1 ▸(*c*)]. The coil periodicity is designed as an integer multiple of the undulator period, which can dramatically reduce the required modulation field strength. The switching mechanism operates by selectively energizing the coils in each undulator. Assume the first undulator is left-hand circularly polarized and the radiation from the second undulator is right-hand circularly polarized. When the coils in the first undulator are activated while those in the second remain off, a red shift occurs in the fundamental energy of the first undulator, and the main flux from the first undulator is shifted out of the monochromator’s bandpass. This leaves only the RCP output from the second undulator, and the radiation from the first undulator has nearly no contribution to the final spectrum. Reversing the activation states of the coils similarly yields pure left-hand circularly polarization, enabling fast switching between polarization states of LCP and RCP. It should be noted that the polarization degree available in this scheme is insensitive to the electron beam emittance or the angular acceptance of the beamline. The radiation performances is easy to predict. This is because, in essence, the radiation from the modulated undulator is shifted almost entirely out of the bandpass of the monochromator. Therefore, the output radiation performances of the system, whether flux or polarization degree, should be the same as those of a conventional unmodulated undulator.

### 2.2. Design of the coils

In the proposed design, the modulation magnetic field is produced by externally introduced electromagnetic coils, the parameter selection of which constitutes a critical aspect of this scheme. These parameters directly determine the achievable modulation field amplitude and, consequently, the induced energy shift of the undulator harmonics. The following section focuses on the methodology for selecting these key parameters.

As established in equation (4), the energy shift depends on the period of the modulation field (represented by the factor *m*) and its strength (represented by α). These correspond, respectively, to the physical dimensions of the electromagnetic coil and the magnetic field it generates. From the equation, it is evident that for a specified energy shift a larger coil period permits a lower strength requirement of magnetic field. To avoid the use of an external water-cooling system, the exciting current density within the coil is strictly limited. For a given conductor cross-section and coil geometry, a larger coil period allows for a greater number of turns. This enables the generation of a stronger magnetic field while maintaining the current density within the maximum permissible limits. Consequently, the primary design task involves the selection of an appropriate coil period, *i.e.* the parameter *m*. This parameter is intrinsically linked to the undulator characteristics, being primarily determined by the number of undulator periods and the undulator strength parameter *K*. Calculation results confirm that a coil satisfying equation (4) under the condition of maximum *K*, which corresponds to the minimum undulator gap and the lowest photon energy, will be capable of generating a sufficient energy shift across the total energy range of the undulator.

**Figure 2:**
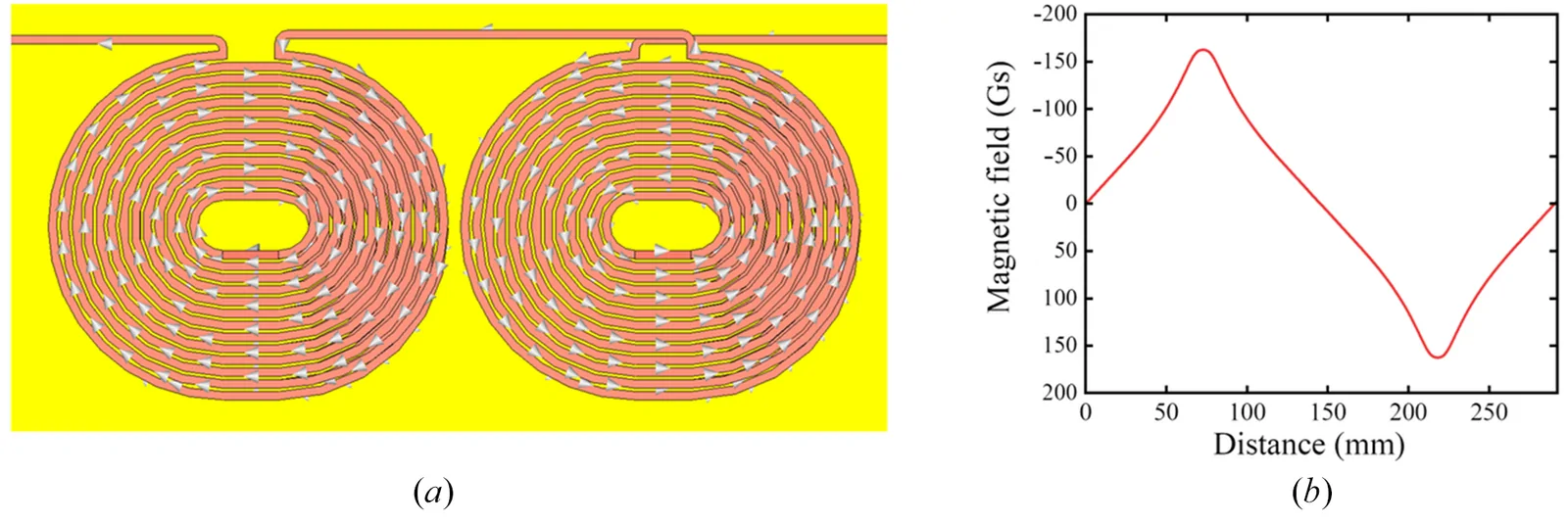
The shape of (*a*) the coils and (*b*) the field distribution.

However, increasing the coil period entails not only a greater longitudinal extent along the electron beam direction but also increased transverse space occupancy (approximately half of the coil period). Therefore, the actual geometry of the vacuum chamber must be considered. This study moves beyond a simplified parallel-plate model (Yang *et al.*, 2024 ▸) and considers the cross-sectional geometry of the vacuum chamber. The chamber features an elliptical beam aperture, with a total thickness of approximately 10 mm and a minimum wall thickness of about 1 mm. The available lateral space for coil installation is constrained to approximately 140 mm. When the coil period exceeds 280 mm, a circular coil becomes impractical. There are two layers in each coil period, assuming using a wire with a cross-sectional size of 1 mm × 1 mm, with each layer of the coil having about 70 turns. A racetrack-type coil geometry, comprising two semicircular arcs connected by straight sections [Fig. 2 ▸(*a*)], is therefore adopted. An example of the magnetic field generated by the coils (*m* = 7) is given in Fig. 2 ▸(*b*) to show the field distribution; the exciting current used in the simulation is 3 A. Furthermore, these coils are designed to correct the first and second integrals of the magnetic field themselves without requiring additional correction magnets, thereby minimizing their impact on the electron beam orbit.

**Figure 3:**
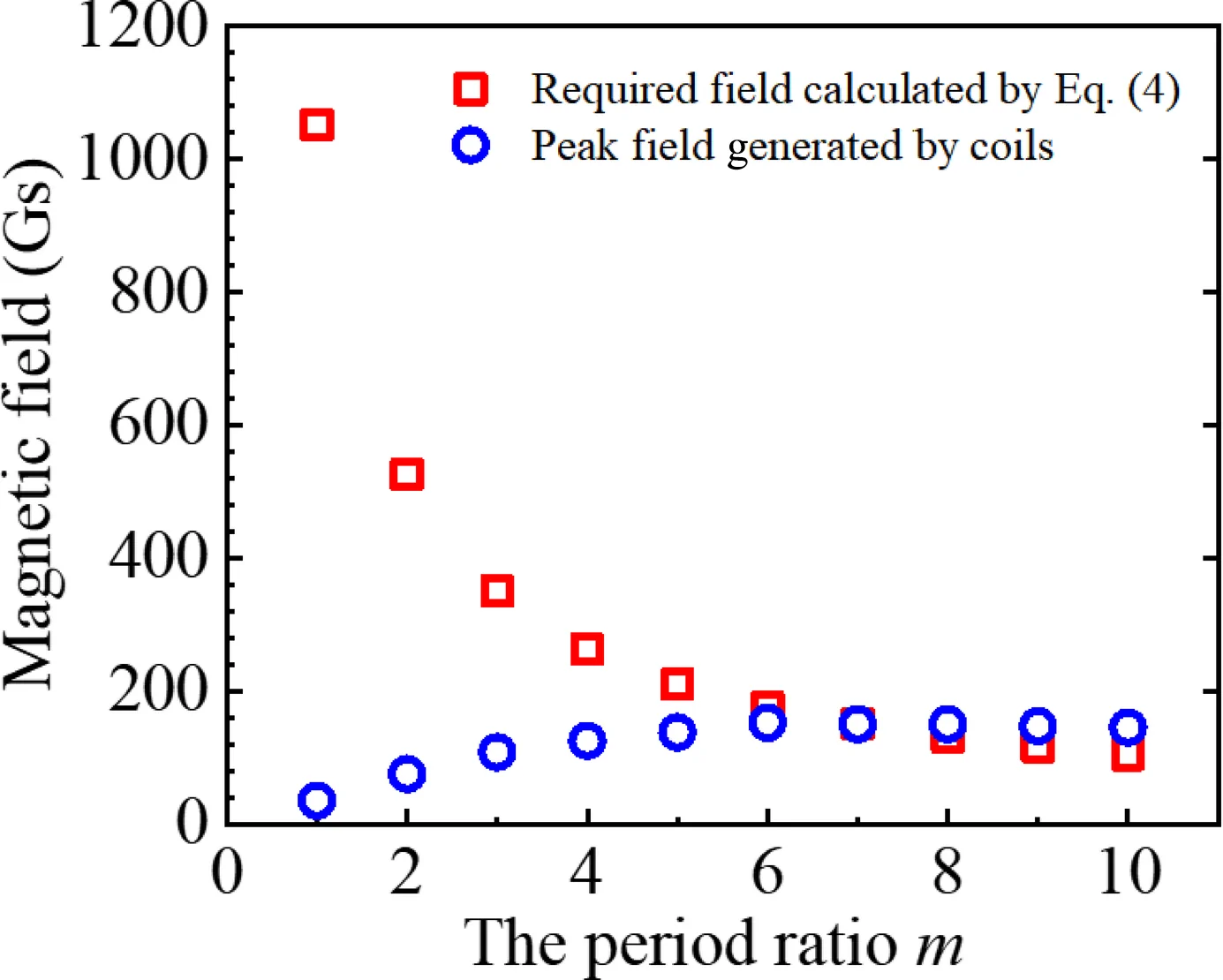
The required magnetic field calculated by equation (4) (red squares) with different period ratio *m* and the peak field the coils can generate (blue circles). The parameters used in the simulations are listed in Table 1 ▸.

The design is based on the parameters of the undulator and storage ring listed in Table 1 ▸. Using equation (4), the required minimum modulation field for various coil periods was calculated (red squares in Fig. 3 ▸). Models of the coils were constructed using *RADIA* (Elleaume *et al.*, 1997 ▸) to determine the maximum achievable magnetic field under the current density limit (blue circles in Fig. 3 ▸).

Considering the cases of *m* = 6 (λ_c_ = 249 mm), *m* = 7 (λ_c_ = 290.5 mm), *m* = 10 (λ_c_ = 415 mm) as examples, the achievable peak fields are almost the same, being approximately 150 Gs. This limitation arises because when *m* exceeds 6 the coils can no longer maintain a circular shape. Consequently, the peak field does not continue to increase as larger coils are employed, while, according to equation (4), 175 Gs, 150 Gs and 103 Gs are required, respectively. Therefore, the period ratio *m* must beat at least 7 to satisfy the requirements of the proposed scheme. For *m* = 7, one fourth of the coil period length is 50 mm. Assuming using the wire with a cross-sectional size of 2 mm × 2 mm, each layer of the coil has about 24 turns.

Actually, if the resonant fundamental photon energy of two undulators is set to be slightly lower than the target photon energy, for example 1.5%–2%, the required coil field can be further reduced. Taking 10-time-period coils as an example, at a target photon energy of 330 eV, the required photon energy shift is approximately 8.3 eV, corresponding to a required coil field of 105 Gs. When an additional −1.5% initial resonant photon energy movement is introduced, the required photon energy shift induced by the coil field is reduced to be approximately 3.3 eV, and the required coil field is approximately 60 Gs. However, in this case, the radiation flux at the target photon energy will lose approximately 60%. When designing and constructing a fast polarization switching beamline, methods to reduce the requirement of modulation magnetic field should be systematically considered based on the practical user requirements.

## 3. Radiation performances

In order to investigate the feasibility of the proposed scheme, we computed the expected performances using the electron beam and undulator parameters listed in Table 1 ▸. In the following numerical study, the radiation at a photon energy of 330 eV is taken as an example and the corresponding undulator strength parameter *K*_*x*_/*K*_*y*_ is 1.55/1.55. In the simulations, the magnetic field is calculated by combining the undulator and the coils as an integrated system using *Radia* and the radiation spectrum is simulated with the *SPECTRA* code (Tanaka & Kitamura, 2001 ▸).

**Figure 4:**
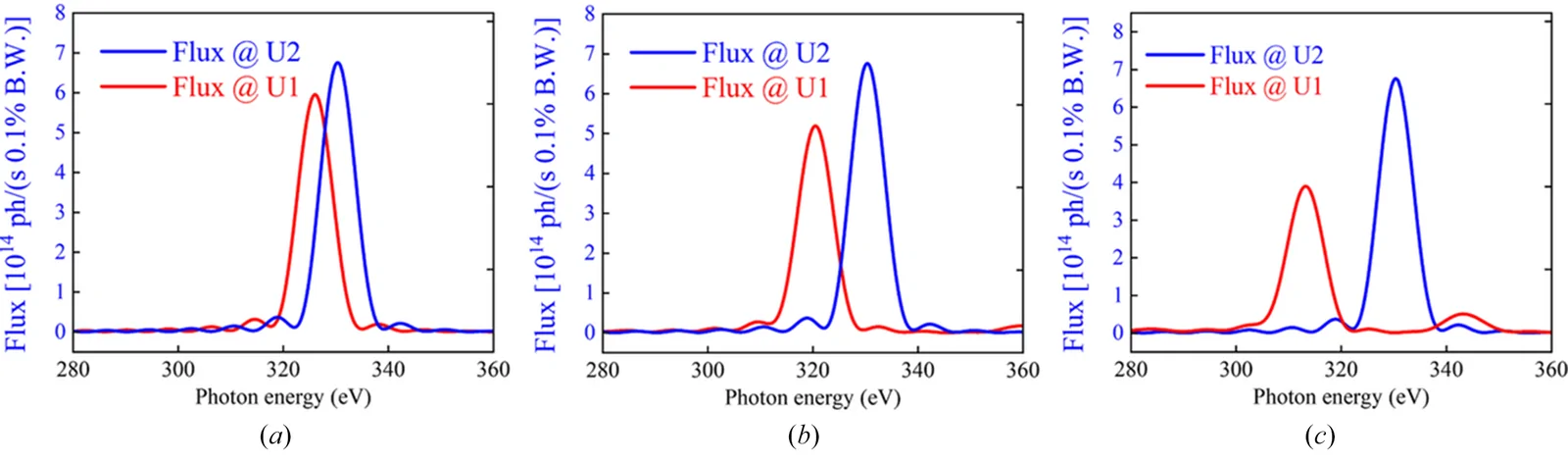
The radiation spectrum modulated by coils (3 A in U2 blue line) with different period ratio: (*a*) *m* = 6, the modulation is not enough to be used in the polarization switching, (*b*) *m* = 7, enough energy shift, (*c*) *m* = 10, energy shift is bigger than that for *m* = 7.

The simulated radiation spectrum is presented in Fig. 4 ▸. At the point of photon energy of 330 eV, which is the fundamental energy of the undulator radiation and also the central energy of the band pass of the monochromator, the radiation flux of the undulator with 7-time-period coils is suppressed to less than 1% of its original value. The flux is further reduced to below 0.1% when coils with a larger period ratio (*m* = 10) are employed.

In our previous work, we examined the influence of vacuum chamber materials on switching frequency, finding that materials with lower electrical conductivity can significantly enhance polarization switching frequency. In similar electromagnetic based schemes, the inductance of the coils and eddy currents are the main issues that limit the switching frequency. We have investigated the main limiting factors for the polarization switching frequency in the proposed scheme. Calculations indicate that the inductance of our system is on the order of tens of µH, while simulations of the entire series-connected coil system confirm that it remains below several hundred µH. These results demonstrate that inductance is not the limiting factor in our design. As mentioned above, for radiation of 330 eV, the coil field should be at least 0.01 T for using the 10-time-period coils. For the coils described in the previous subsection, the inductance of each coil is about several µH, which has a negligible impact on the polarization switching frequency. Instead, the primary constraint is the field penetration delay caused by eddy currents as the magnetic field penetrates the vacuum chamber wall.

**Figure 5:**
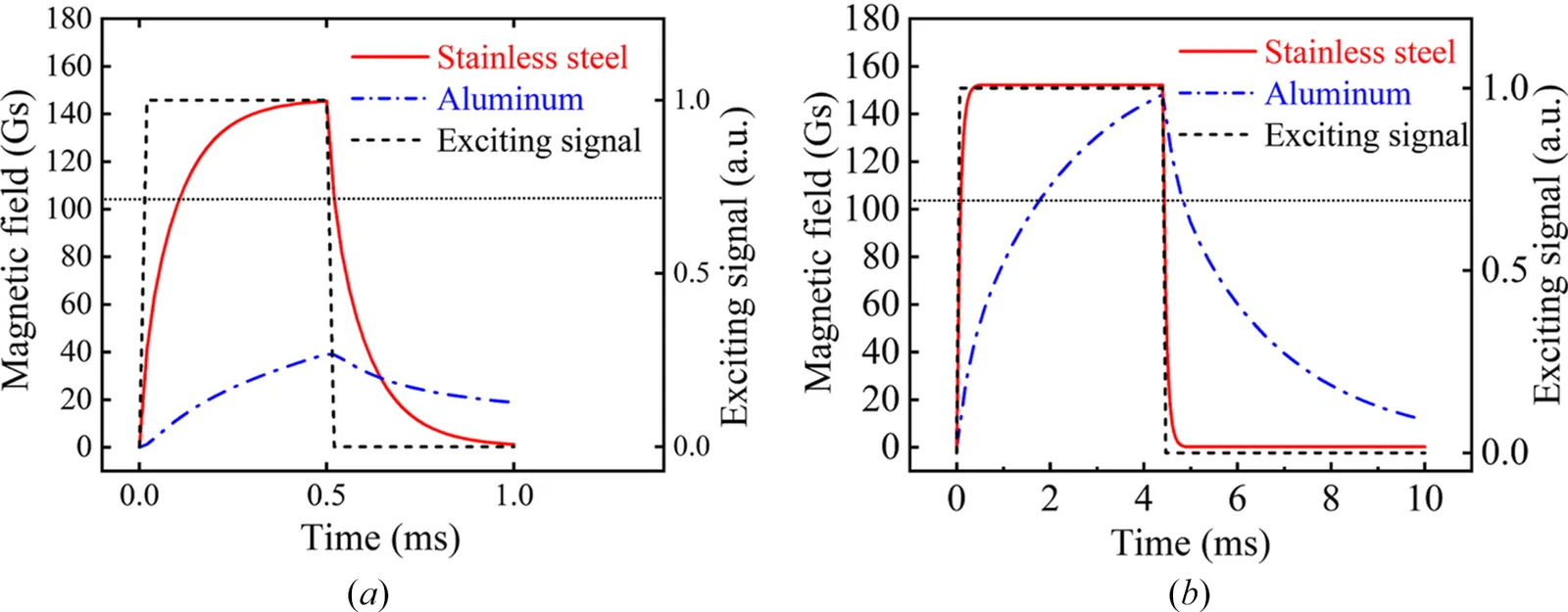
The square wave exciting signal (black dashed line) and the response of the magnetic field on the beam orbit for stainless steel vacuum chamber (red solid line) and aluminium vacuum chamber (blue dash-dotted line) with actual shape using 10-time-period coils. The periods of the square wave exciting signal are (*a*) 1 ms and (*b*) 10 ms.

Fig. 5 ▸ illustrates how different kinds of vacuum chamber material physically influence the pulsed magnetic field of the coils. The impact of the vacuum chamber material as well as the inductance of the coils on the time delay of the pulsed magnetic field has been evaluated employing the *CST Studio Suite*. First, we focus on the results of the proposed scheme in the HALF vacuum chamber, an aluminium vacuum chamber. A square-wave exciting current pulse with width of about 4.5 ms was used to feed the coils as an example. The field response on the beam orbit of the coil can meet the requirement of several hundreds hertz switching frequency. Under these conditions, if the peak magnetic field exceeds 100 Gs, it is sufficient to generate enough photon energy shift. For a stainless steel vacuum chamber, as shown in Fig. 5 ▸(*a*), the results demonstrate that the rise and fall process of the coil field can be completed in less than 0.5 ms for each current pulse, which means fast polarization switching at the frequency of kilohertz can be expected.

**Figure 6:**
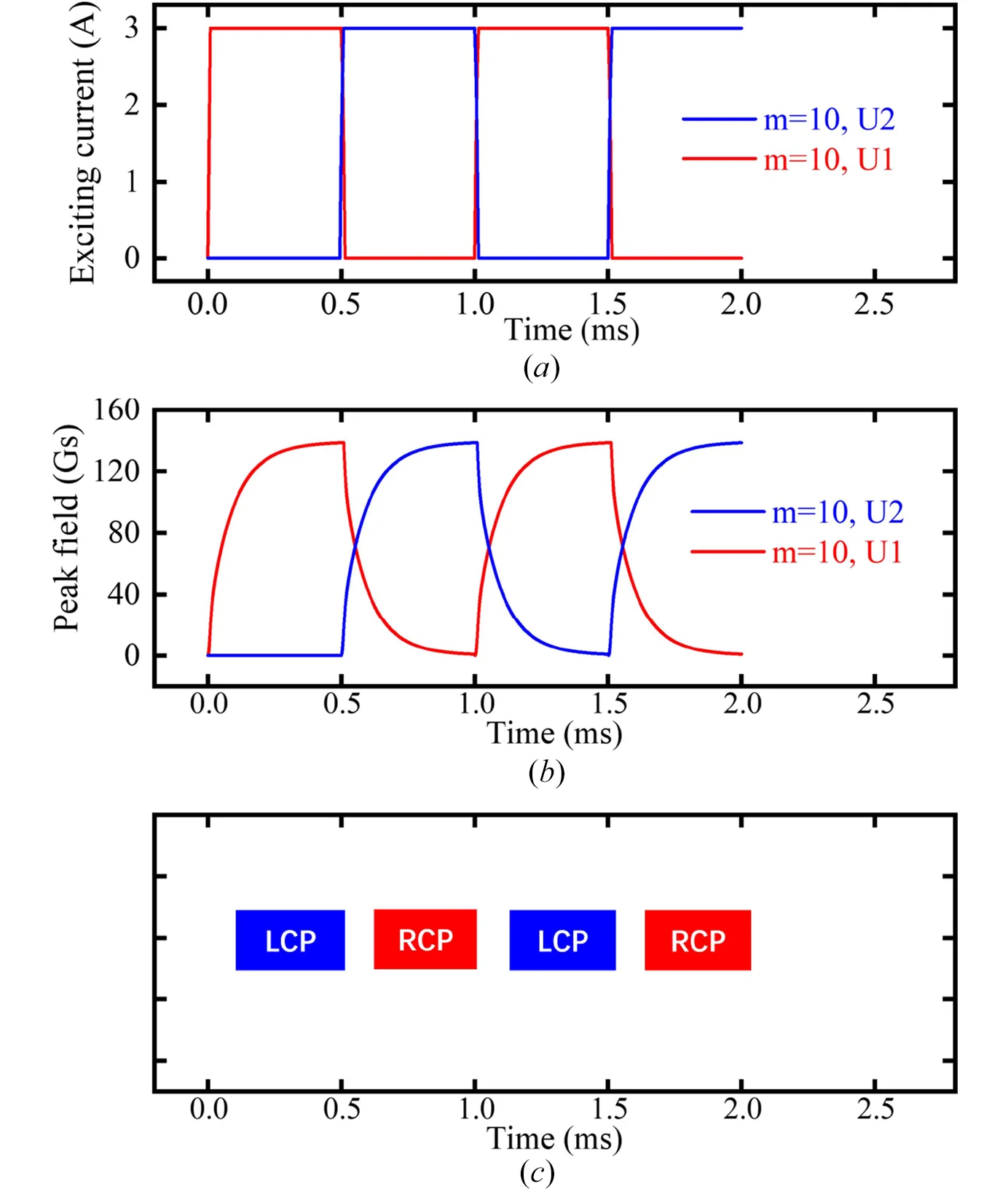
(*a*) The square wave exciting signal in the first undulator (U1, red line) and in the second undulator (U2, blue line). (*b*) The response of the magnetic field on the beam orbit with *m* = 10 in the first undulator (U1, red line) and in the second undulator (U2, blue line) and using stainless steel vacuum chamber. (*c*) The final polarization state after a monochromator in polarization switching.

Using *CST*, coils with *m* = 10 under realistic vacuum chamber structure are selected as an example to show the exciting current, magnetic field response of the coils and the polarization switching. In the simulation, the vacuum chamber material was chosen as stainless steel. The results are shown in Fig. 6 ▸. Fig. 6 ▸(*a*) shows the exciting current used in the simulation. Fig. 6 ▸(*b*) presents the magnetic field response of the electromagnetic coils. Fig. 6 ▸(*c*) shows the polarization state of the radiation after passing through the monochromator. For example, when the magnetic field of the electromagnetic coil in U1 rises to above 105 Gs, the radiation from U1 is shifted out of the band pass of the monochromator, and the polarization state of the radiation after the monochromator is the same with the radiation from U2.

The field response of the coils using the aluminium vacuum chamber has also been simulated. As shown in Fig. 5 ▸(*b*), the field can only reach 38 Gs in 0.5 ms, which means that an aluminium vacuum chamber cannot meet the requirements of achieving a kHz-level polarization switching.

## 4. Summary

The proposed kHz-level polarization switching scheme for the Hefei Advanced Light Facility introduces an innovative magnetic field modulation technique to enable fast switching between left-hand and right-hand circularly polarized undulator radiation. The system employs two helical undulators with opposite helicities, each equipped with electromagnetic coils that modulate the undulator spectrum when excited, thereby detuning harmonics and suppressing unwanted polarization states to below 1% intensity. The method allows a downstream monochromator to effectively select the desired polarization state, achieving clean, fast polarization switching, critical for experiments like X-ray magnetic circular dichroism. The key to this design is an optimized coil geometry with a periodicity multiple (*e.g. m* = 10) of the undulator period, reducing the required modulation field strength. Simulations confirm effective photon energy shifts and radiation performances with high flux and polarization degree. Field response times under 0.5 ms support the feasibility of kHz-level polarization switching. Consequently, the proposed scheme provides a viable and high-performance solution for the HALF XMCD experiments.

## Figures and Tables

**Table 1:** Parameters used in simulations

Parameter	Value	Unit
Electron energy	2.2	GeV
Average beam current	350	mA
Electron beam emittance	100	pm rad
Undulator period λ_u_	41.5	mm
Number of periods of each undulator *N*_u_	41	–
Undulator parameter *K*_*x*_ / *K*_*y*_	1.55 / 1.55	–
Photon energy	330	eV
Exciting current density	3	A mm^−2^

## References

[bb1] Bahrdt, J., Frentrup, W., Gaupp, A., Scheer, M., Gudat, W., Ingold, G. & Sasaki, S. (2001). *Nucl. Instrum. Methods Phys. Res. A* **467–468**, 21–29.

[bb2] Bahrdt, J., Gaupp, A., Gudat, W., Mast, M., Molter, K., Peatman, W. B., Scheer, M., Schroeter, T. & Wang, C. (1992). *Rev. Sci. Instrum.* **63**, 339–342.

[bb3] Bai, Z. H., Liu, G. W., He, T. L., Wang, L., Hu, N. & Li, H. T. (2021). *Proceedings of IPAC2021* pp. 407–409, Campinas, SP, Brazil.

[bb4] Cao, J., Wang, Y., Zou, Y., Zhang, X., Wu, Y. & Tai, R. (2016). *J. Synchrotron Rad.* **23**, 436–442. 10.1107/S160057751600059X 26917130

[bb5] Chubar, O., Benabderrahmane, C., Marcouille, O., Marteau, F., Massal, M. & Veteran, J. (2004). *Proceedings of EPAC2004*pp. 1675–1677, Lucerne, Switzerland.

[bb671] Deng, H., Zhang, T., Feng, L., Feng, C., Liu, B., Wang, X., Lan, T., Wang, G., Zhang, W., Liu, X., Chen, J., Zhang, M., Lin, G., Zhang, M., Wang, D. & Zhao, Z. (2014). *Phys. Rev. ST Accel. Beams*, **17**, 020704.

[bb6] Elleaume, P., Chubar, O. & Chavanne, J. (1997). *Proceedings of PAC1997* pp. 3509–3511, Vancouver, BC, Canada.

[bb7] Hara, T., Shirasawa, K., Takeuchi, M., Seike, T., Saito, Y., Muro, T. & Kitamura, H. (2003). *Nucl. Instrum. Methods Phys. Res. A* **498**, 496–502.

[bb8] Holldack, K., Schüssler-Langeheine, C., Goslawski, P., Pontius, N., Kachel, T., Armborst, F., Ries, M., Schälicke, A., Scheer, M., Frentrup, W. & Bahrdt, J. (2020). *Commun. Phys.* **3**, 61.

[bb9] Kaneyasu, T., Hikosaka, Y., Fujimoto, M., Iwayama, H. & Katoh, M. (2020). *New J. Phys.* **22**, 083062.

[bb10] Kesgin, I., Ivanyushenkov, Y., Kasa, M., Leighty, B. & Hasse, Q. (2019). *Proceedings of NAPAC2019*, pp. 497–499, Lansing, MI, USA.

[bb11] Kim, K. J. (1984). *Nucl. Instrum. Methods Phys. Res.* **219**, 425–429.

[bb12] Kinjo, R. & Tanaka, T. (2016). *J. Synchrotron Rad.* **23**, 751–757. 10.1107/S1600577516004604 PMC5356622 27140155

[bb13] Liang, X., Calvi, M., Couprie, M. E., Ganter, R., Kittel, C., Sammut, N., Schmidt, T., Valléau, M. & Zhang, K. (2021). *Nucl. Instrum. Methods Phys. Res. A* **987**, 164741.

[bb14] Marteau, F., Berteaud, P., Bouvet, F., Brunelle, P., Chubar, O. & Vétéran, J. (2009). *Proceedings of PAC2009* pp. 2456–2458, Vancouver, BC, Canada.

[bb15] Marteau, F., Berteaud, P., Bouvet, F., Brunelle, P., Chubar, O. & Vétéran, J. (2011). *Proceedings of IPAC2011* pp. 3239–3241, San Sebastián, Spain.

[bb16] Matsuda, I., Kuroda, A., Miyawaki, J., Kosegawa, Y., Yamamoto, S., Seike, T., Bizen, T., Harada, Y., Tanaka, T. & Kitamura, H. (2014). *Nucl. Instrum. Methods Phys. Res. A* **767**, 296–299.

[bb17] Miyawaki, J., Yamamoto, S., Hirata, Y., Horio, M., Harada, Y. & Matsuda, I. (2021). *AAPPS Bull.* **31**, 25.

[bb18] Rowen, M. & Lüning, J. (2001). *Nucl. Instrum. Methods Phys. Res. A* **467–468**, 169–172.

[bb25] Sánchez-Hanke, C., Kao, C. C. & Hulbert, S. (2009). *Nucl. Instrum. Methods Phys. Res. A* **608**, 351–359.

[bb19] Sasaki, S. (1994). *Nucl. Instrum. Methods Phys. Res. A* **347**, 83–86.

[bb20] Sasaki, S. (2010). *Proceedings of IPAC10*, pp. 3141–3143, Kyoto, Japan.

[bb21] Sasaki, S., Kakuno, K., Takada, T., Shimada, T., Yanagida, K. & Miyahara, Y. (1993). *Nucl. Instrum. Methods Phys. Res. A* **331**, 763–767.

[bb22] Sawhney, K., Senf, F., Scheer, M., Schäfers, F., Bahrdt, J., Gaupp, A. & Gudat, W. (1997). *Nucl. Instrum. Methods Phys. Res. A* **390**, 395–402.

[bb23] Schmidt, T. & Calvi, M. (2018). *Synchrotron Radiat. News* **31**, 35–40.

[bb24] Schmidt, T., Ingold, G., Imhof, A., Patterson, B. D., Patthey, L., Quitmann, C., Schulze-Briese, C. & Abela, R. (2001). *Nucl. Instrum. Methods Phys. Res. A* **467–468**, 126–129.

[bb26] Tanaka, T., Hara, T., Oura, M., Ohashi, H., Kimura, H., Goto, S., Suzuki, Y. & Kitamura, H. (1999). *Rev. Sci. Instrum.* **70**, 4153–4160.

[bb27] Tanaka, T. & Kitamura, H. (1995). *Nucl. Instrum. Methods Phys. Res. A* **364**, 368–373.

[bb28] Tanaka, T. & Kitamura, H. (2001). *J. Synchrotron Rad.* **8**, 1221–1228. 10.1107/s090904950101425x 11679776

[bb29] Tanaka, T. & Kitamura, H. (2002). *Nucl. Instrum. Methods Phys. Res. A* **490**, 583–591.

[bb30] Tanaka, T. & Kitamura, H. (2004). *AIP Conf. Proc.* **705**, 231–234.

[bb31] Tanaka, T., Shirasawa, K. & Kitamura, H. (2002). *Rev. Sci. Instrum.* **73**, 1724–1727.

[bb32] Tsuchiya, K., Shioya, T., Aoto, T., Harada, K., Obina, T., Sakamaki, M. & Amemiya, K. (2013). *J. Phys. Conf. Ser.* **425**, 132017.

[bb33] Wang, L., Bai, Z. H., Hu, N., Li, H. T., He, T. L. & Liu, G. W. (2018). *Proceedings of IPAC2018* pp. 4598–4600, Vancouver, BC, Canada.

[bb34] Weiss, M. R., Follath, R., Sawhney, K. J. S., Senf, F., Bahrdt, J., Frentrup, W., Gaupp, A., Sasaki, S., Scheer, M., Mertins, H., Abramsohn, D., Schäfers, F., Kuch, W. & Mahler, W. (2001). *Nucl. Instrum. Methods Phys. Res. A* **467–468**, 449–452.

[bb35] Yamamoto, S., Senba, Y., Tanaka, T., Ohashi, H., Hirono, T., Kimura, H., Fujisawa, M., Miyawaki, J., Harasawa, A., Seike, T., Takahashi, S., Nariyama, N., Matsushita, T., Takeuchi, M., Ohata, T., Furukawa, Y., Takeshita, K., Goto, S., Harada, Y., Shin, S., Kitamura, H., Kakizaki, A., Oshima, M. & Matsuda, I. (2014). *J. Synchrotron Rad.* **21**, 352–365. 10.1107/S1600577513034796 PMC3945419 24562556

[bb36] Yang, N., Xu, Y., Zhao, Z. & Li, H. (2023). *Phys. Rev. Accel. Beams* **26**, 080701.

[bb37] Yang, N., Zhao, Z. & Li, H. (2024). *Phys. Rev. Accel. Beams* **27**, 070702.

[bb38] Zhao, Z., Xu, Y. & Li, H. (2023). *Proceedings of IPAC2023* pp. 1247–1249, Venice, Italy. MOPM115

